# Supplementary material for: The causal role of circulating inflammatory markers in osteoporosis: a bidirectional Mendelian randomized study
Source: Front Immunol. 2024 Jul 18;15:1412298. doi: 10.3389/fimmu.2024.1412298 (PMC11291241; doi:10.3389/fimmu.2024.1412298)
Supplement: Supplementary file 1 [file DataSheet_1.zip › supplementary material/OSTEOPOROSIS-CIM/GCST90274776.tsv.gz_forest.pdf]

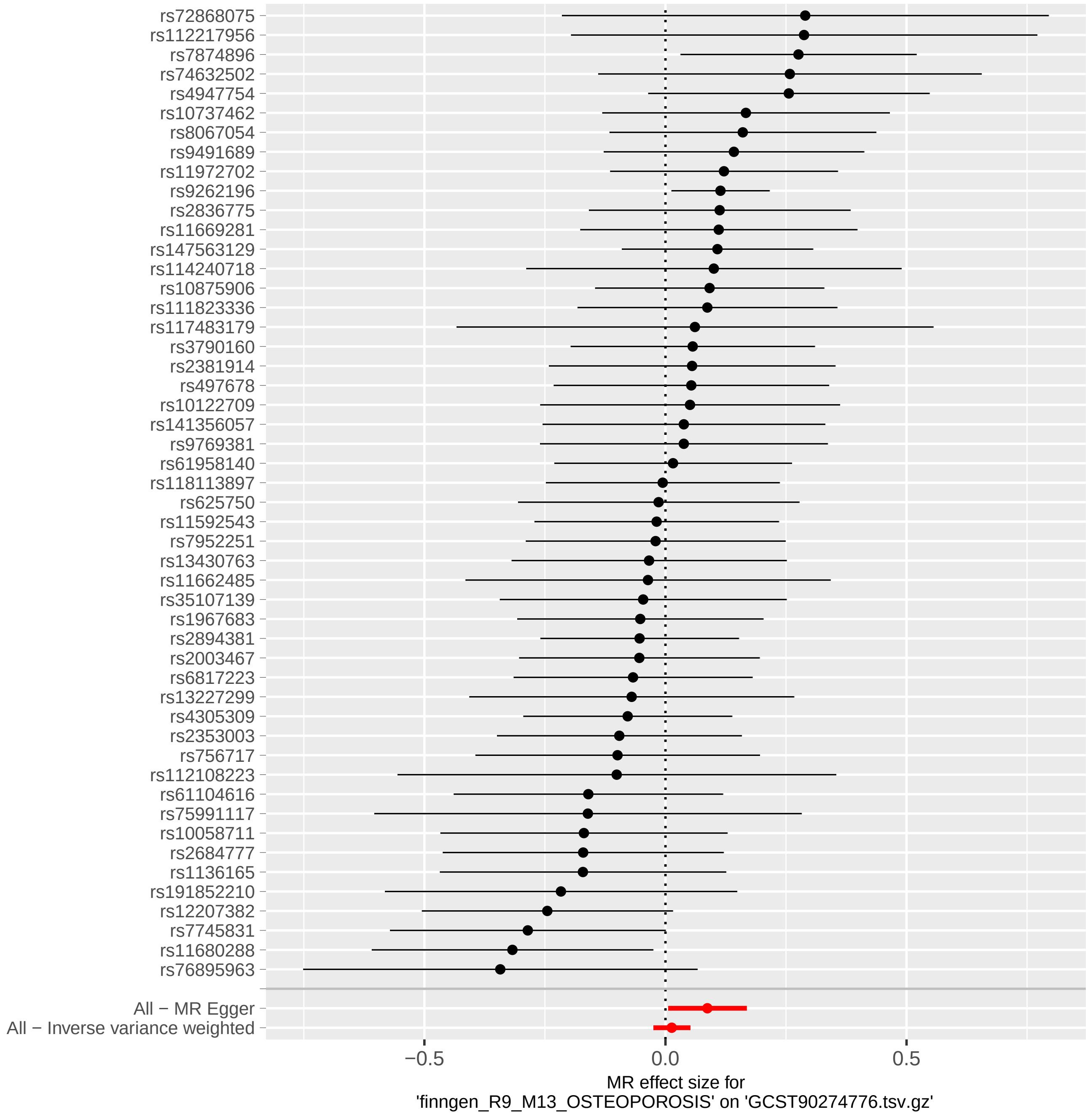
rs72868075
rs112217956
rs7874896
rs74632502
rs4947754
rs10737462
rs8067054
rs9491689
rs11972702
rs9262196
rs2836775
rs11669281
rs147563129
rs114240718
rs10875906
rs111823336
rs117483179
rs3790160
rs2381914
rs497678
rs10122709
rs141356057
rs9769381
rs61958140
rs118113897
rs625750
rs11592543
rs7952251
rs13430763
rs11662485
rs35107139
rs1967683
rs2894381
rs2003467
rs6817223
rs13227299
rs4305309
rs2353003
rs756717
rs112108223
rs61104616
rs75991117
rs10058711
rs2684777
rs1136165
rs191852210
rs12207382
rs7745831
rs11680288
rs76895963
All − MR Egger
All − Inverse variance weighted
−0.5
0.0
0.5
MR effect size for
'finngen_R9_M13_OSTEOPOROSIS' on 'GCST90274776.tsv.gz'
